# An Evidence Map of the Women Veterans’ Health Research Literature (2008–2015)

**DOI:** 10.1007/s11606-017-4152-5

**Published:** 2017-09-14

**Authors:** Elisheva R. Danan, Erin E. Krebs, Kristine Ensrud, Eva Koeller, Roderick MacDonald, Tina Velasquez, Nancy Greer, Timothy J. Wilt

**Affiliations:** 1 VA HSR&D Center for Chronic Disease Outcomes Research, Minneapolis VA Healthcare System, 1 Veterans Drive (152), Minneapolis, MN 55417 USA; 2 Department of Medicine, University of Minnesota Medical School, Minneapolis, MN USA

## Abstract

**Background:**

Women comprise a growing proportion of Veterans seeking care at Veterans Affairs (VA) healthcare facilities. VA initiatives have accelerated changes in services for female Veterans, yet the corresponding literature has not been systematically reviewed since 2008. In 2015, VA Women’s Health Services and the VA Women’s Health Research Network requested an updated literature review to facilitate policy and research planning.

**Methods:**

The Minneapolis VA Evidence-based Synthesis Program performed a systematic search of research related to female Veterans’ health published from 2008 through 2015. We extracted study characteristics including healthcare topic, design, sample size and proportion female, research setting, and funding source. We created an evidence map by organizing and presenting results within and across healthcare topics, and describing patterns, strengths, and gaps.

**Results:**

We identified 2276 abstracts and assessed each for relevance. We excluded 1092 abstracts and reviewed 1184 full-text articles; 750 were excluded. Of 440 included articles, 208 (47%) were related to mental health, particularly post-traumatic stress disorder (71 articles), military sexual trauma (37 articles), and substance abuse (20 articles). The number of articles addressing VA priority topic areas increased over time, including reproductive health, healthcare organization and delivery, access and utilization, and post-deployment health. Three or fewer articles addressed each of the common chronic diseases: diabetes, hypertension, depression, or anxiety. Nearly 400 articles (90%) used an observational design. Eight articles (2%) described randomized trials.

**Conclusions:**

Our evidence map summarizes patterns, progress, and growth in the female Veterans’ health and healthcare literature. Observational studies in mental health make up the majority of research. A focus on primary care delivery over clinical topics in primary care and a lack of sex-specific results for studies that include men and women have contributed to research gaps in addressing common chronic diseases. Interventional research using randomized trials is needed.

## INTRODUCTION

Despite serving in or alongside the US military since the Revolutionary War, women have experienced unequal access to Veterans Affairs (VA) benefits, and few women used the VA healthcare system prior to the early 1980s.^1^ In the subsequent 30 years, clinical, research, and policy initiatives have sought to improve the quality and accessibility of evidence-based healthcare for female Veterans.^2^ Today, women are the fastest-growing population of US Veterans receiving VA healthcare.^3^

When the literature related to female Veterans’ health and healthcare was last reviewed in 2008,^4^^–^ 6 the authors encountered a rapidly emerging field of research. They described growth in research related to access, utilization, and organizational quality, but identified gaps in research related to chronic physical and mental health conditions, complex combinations of disease, pregnancy and aging, traumatic brain injury, co-managed mental and physical preventive care, and post-deployment transitional health. Subsequently, the VA women’s health landscape has changed substantially. In 2008, the national Women’s Health Services (WHS) program was established to oversee clinical initiatives, such as the provision of comprehensive women’s healthcare (including general and gender-specific care) at a single site from a single provider.^3^ The VA Women’s Health Research Network (WHRN) was created in 2010 to fill knowledge gaps in the evidence base related to female Veterans’ health and healthcare.^7^ Based in part on the results of the previous review,^5^ the WHRN prioritized research on six key topic areas: (1) mental health, (2) primary care and prevention, (3) reproductive health, (4) complex chronic conditions/aging and long-term care, (5) access to care and rural health, and (6) post-deployment health.^8^

In this paper, we present an evidence map of the existing literature related to female Veterans’ health and healthcare published from 2008 through 2015, based on a VA Evidence-based Synthesis Program (ESP) report available at http://vaww.hsrd.research.va.gov/publications/esp/womens-health2.cfm. This review was requested jointly by VA WHS and the VA WHRN.

## METHODS

Evidence maps identify and organize the existing literature within a broad subject area to facilitate future research and policy planning. Given the interval growth and expansive scope of the literature related to female Veterans’ health and healthcare, we elected to create an evidence map rather than perform a traditional systematic review. A systematic review typically addresses a specific research question within a narrowly defined population. Our operational partners in the clinical and research offices of women’s health at the VA asked us to instead describe all facets of the female Veterans’ health literature. Key features of an evidence map include early involvement of stakeholders, a systematic search strategy, and a visual representation that presents the identified literature.^9^ Evidence maps do not involve assessing study quality or risk of bias, or extracting, evaluating, or synthesizing study findings.^10^ We describe multiple characteristics of the literature but provide a limited assessment of research quality.

### Data Sources and Searches

We searched MEDLINE (Ovid), the Cumulative Index to Nursing and Allied Health Literature (CINAHL), and the VA Health Services Research and Development database for articles published between January 2008 and December 2015. The previous review period ended in September 2008, allowing a short overlap period to capture pending or unindexed publications. The search included the Medical Subject Headings (MeSH) terms Women; Women’s Health; Women’s Health Services; Veterans; Veterans Health; and Hospitals, Veterans.

### Study Selection

We excluded studies that were not relevant to health/healthcare, did not include female US Veterans, or only included active duty military. Studies with fewer than 100 participants were excluded if less than 10% of participants were women, and studies with 100 to 1000 participants were excluded if less than 5% were women. Studies with more than 1000 participants were eligible if they included any women. For studies with a female or Veteran proportion < 75% of the total study population, we excluded studies that did not stratify results by sex or Veteran status, respectively. We also excluded case reports, letters, meeting abstracts, dissertations, editorials, reviews, conceptual frameworks, and protocols.

Abstracts were independently reviewed by a trained investigator (ED and NG) or research associate (EK, TV, and RM). A random selection of 18% (404 abstracts) were dual-reviewed; for these, agreement on inclusion was 87% (κ = 0.747), which is considered by convention to be substantial or moderate agreement.^11^ The full texts of eligible studies were then independently reviewed for inclusion by an investigator or research associate. A second reviewer independently reviewed a 10% random sample of full-text articles, as well as any additional articles that the original reviewer requested. If the two reviewers disagreed, a group arbitration system was used.

### Data Abstraction

For each included study, 15 study characteristics were extracted onto evidence tables by one investigator or research associate. We selected and defined these study characteristics after discussion with key stakeholders in the clinical and research offices of women’s health at the VA and an expert panel composed of VA women’s healthcare providers and researchers, and then refined the categories within each study characteristic through multiple small subsample extractions. The characteristics extracted were healthcare topic, study design, sample size and proportion female, reporting of age and race, focus on special populations (e.g., lesbian, gay, bisexual, and transgender (LGBT), racial and ethnic minorities, or homeless Veterans), follow-up/duration, research setting, use of electronic health record, period of service, Veteran engagement (i.e., participation of patients in the design or conduct of the study), population studied, type of outcomes reported, publication year, and funding source. Each included study was designated one of 39 healthcare topics based on the primary focus of the article, and we grouped these under four subheadings (Table 1). Articles that reported on physical or mental health topics but primarily addressed issues of prevention and screening, healthcare organization and delivery, access and utilization, homelessness, or post-deployment health were placed in the latter groupings. We then performed an iterative, cross-tabular review of the abstracted data. A randomly selected 10% sample of studies were individually dual-reviewed across each column of study characteristics. Inter-rater discrepancies were noted within particular columns that relied on subjective interpretation. We addressed discrepancies by either reducing category granularity (e.g., we collapsed all observational studies except prospective cohort studies into a single grouping) or assigning two researchers to dual review all the studies grouped within a particular category, in order to ensure consistent assignment within each column. The principal investigator verified categorizations and addressed inconsistencies while summarizing the findings by study characteristics.

**Table 1:** Healthcare Topics

Healthcare Topics		Number of Studies
Mental Health *Total: 208 articles*	PTSD and trauma	71
	Military sexual trauma	37
	Mental health comorbid with non-mental health	23
	Substance abuse	20
	Multiple mental health diagnoses	16
	Suicide	13
	Intimate partner violence	9
	Disordered eating	5
	Depression and anxiety	4
	Reproductive mental health	4
	Serious mental illness	3
	Personality disorders	0
	Other mental health topics	3
Physical Health *Total: 133 articles*	Reproductive health	24
	Prevention/Screening	18
	Long-term care/aging	13
	Cardiovascular disease	11
	Obesity	9
	Chronic pain	7
	Comorbid medical conditions	7
	Cancer	6
	Tobacco	6
	Traumatic brain injury	5
	HIV/AIDS	5
	Multiple sclerosis	4
	Diabetes	3
	Spinal cord injury	1
	Traumatic amputations	1
	Hypertension	0
	Other medical conditions	13
Healthcare Organization and Delivery *Total: 31 articles*	Comprehensive and primary care delivery	16
	Mental healthcare delivery	9
	Emergency care delivery	3
	Virtual or telehealthcare delivery	3
Access, Utilization & Post-Deployment Health *Total: 57 articles*	Post-deployment health	18
	Barriers and facilitators of care	13
	Homelessness	12
	Healthcare utilization	11
	Rural healthcare	3
Other		11
TOTAL NUMBER OF INCLUDED STUDIES		440

### Data Synthesis and Analysis

We sorted and compared studies by healthcare topic, study design, sample size and proportion female, publication year, and funding source.

## RESULTS OF LITERATURE SEARCH

**Fig. 1:**
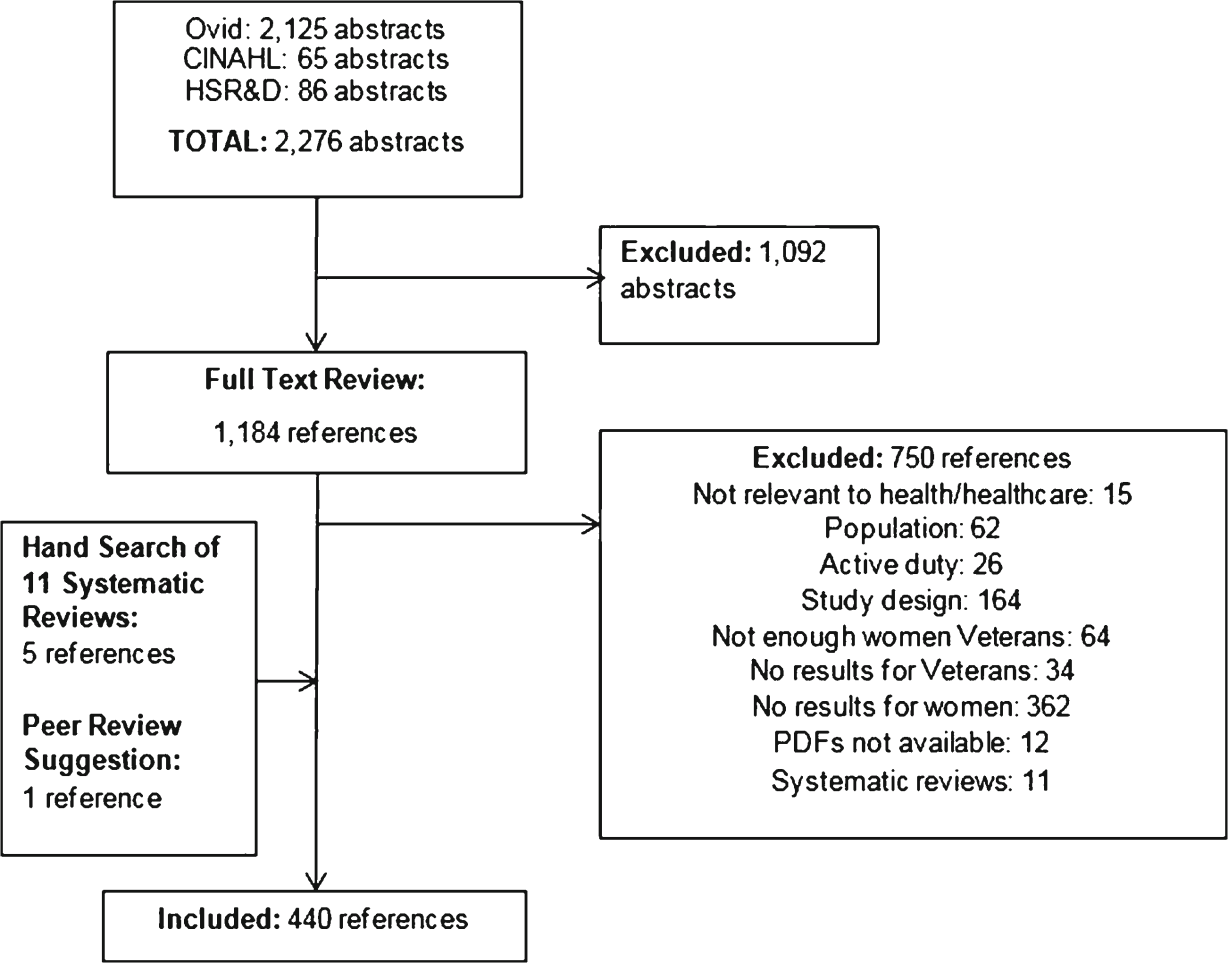
Literature Flow Chart.

We reviewed 2276 abstracts, excluded 1092, and reviewed the full text of 1184 references (Fig. 1). During full-text review we excluded 750 articles, leaving 434 eligible for inclusion. An additional six studies not found by our literature search were identified by searching references of 11 systematic reviews or during peer review of the draft report, bringing the total number of included references to 440 (Appendix A).

## SUMMARY OF RESULTS

**Fig. 2:**
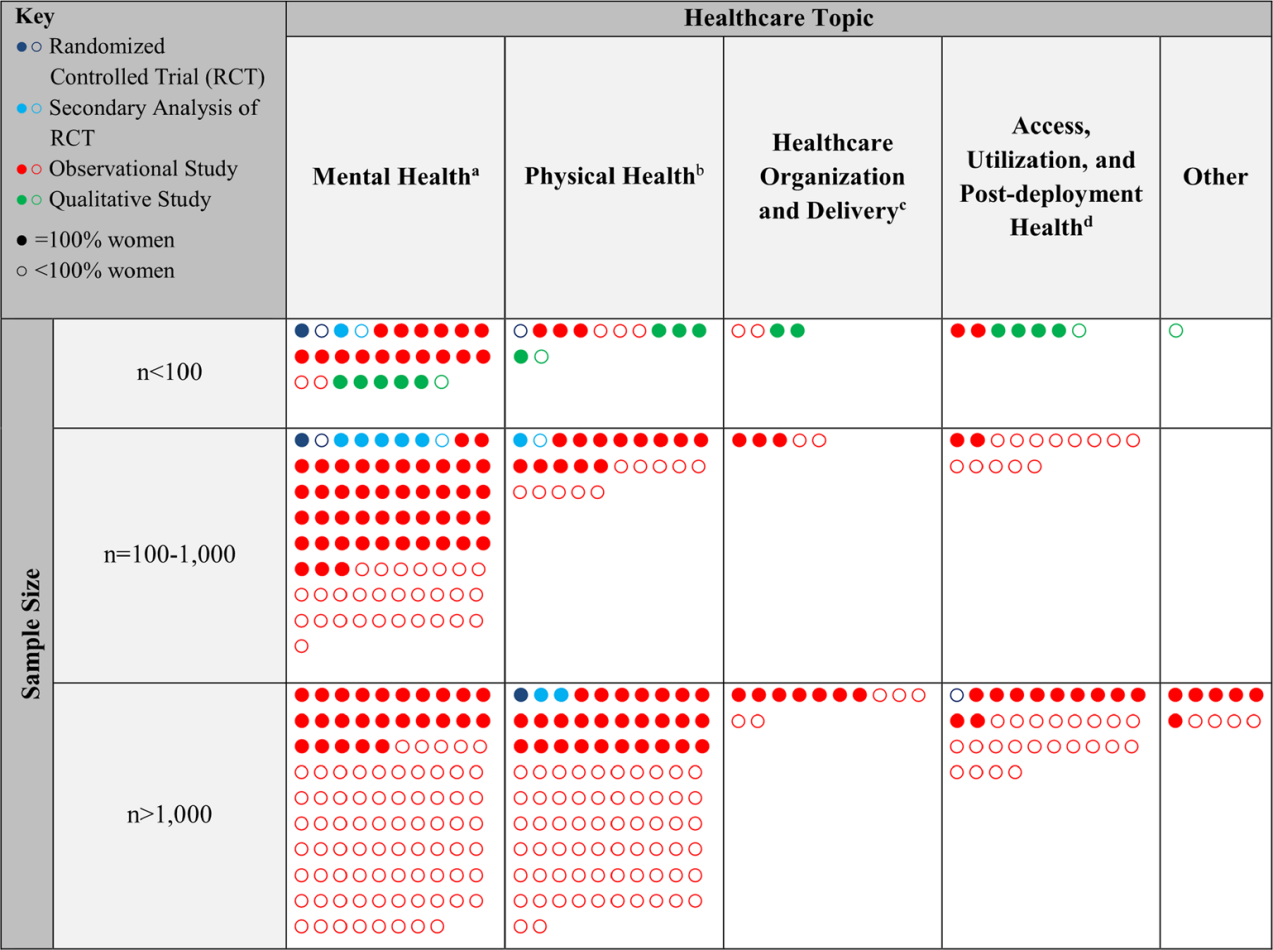
Included Studies by Healthcare Topic, Sample Size and Proportion Female, and Study Design. ^a^One additional observational study of facilities; size of study and % women not applicable. ^b^ One additional observational study with *n* > 1000; % women not reported; Two additional observational studies with *n* = 100–1000 and one observational study with *n* < 100; % women not applicable. ^c^10 additional studies: % women not applicable for 1 RCT/CCT (*n* = 100–1000), 5 observational studies (2 with *n* = 100–1000, 3 with *n* < 100) and 3 qualitative studies (all *n* < 100); % women not reported for 1 observational study (*n* > 1000). ^d^One additional observational study with *n* > 1000; % women not reported.

An overall visual representation of the included studies by healthcare topic subheading, sample size and proportion female, and study design is presented in Figure 2.

*Healthcare topic:* Most studies were related to mental health (208/440, 47%) or physical health conditions (133/440, 30%; Table 1).

### Mental Health Conditions

Mental health articles were dominated by conditions often associated with military service, primarily post-traumatic stress disorder (PTSD) (71/208, 34%), military sexual trauma (MST) (37/208, 18%), and substance abuse (20/208, 10%). Four observational studies primarily addressed depression (3) and anxiety (1), and eight others addressed depression comorbid with other mental or physical health conditions. Four articles described reproductive mental health issues (e.g., postpartum depression). Twelve articles presented the primary findings (4 studies) or secondary analyses (8 studies) of randomized trials related to PTSD, MST, or multiple mental health diagnoses.

### Physical Health Conditions

No specific clinical condition dominated the physical health articles, and few articles were found regarding common chronic conditions such as obesity (9), chronic pain (7), diabetes (3), and hypertension (0). The four most common topics were prevention and screening, reproductive health, long-term care and aging, and cardiovascular disease; together, they made up half (66/133, 50%) of the physical health articles. Though most studies were observational, there was one randomized controlled trial (RCT) on mammography screening promotion among women Veterans published in 2008,^12^ three subsequent secondary analyses of that study, and one small, single-site 6-month RCT of aerobic exercise for mild cognitive impairment.^13^

### Healthcare Organization and Delivery

Thirty-one studies evaluated healthcare organization and delivery, 45% of which were published in 2015. These studies described the challenges, methods, and outcomes related to healthcare delivery for female Veterans. Half focused on comprehensive primary care for female Veterans, including a single VA-funded RCT of VA healthcare providers that tested the effects of a 30-min computerized educational program on gender awareness.^14^ We identified nine studies related to mental healthcare delivery for female Veterans, and three studies each related to emergency care delivery and virtual or telehealthcare delivery methods.

### Access, Utilization, and Post-Deployment Health

We identified 57 articles related to access and utilization (24), rural healthcare (3), homelessness (12), or post-deployment health (18). The access and utilization studies assessed barriers to care related to homelessness, mental healthcare, financial concerns, and factors that explain delayed care and attrition, and described VA and non-VA healthcare utilization. Over a third of these specifically addressed Veterans of Iraq and Afghanistan conflicts (9/24, 38%). Nearly half of the 18 studies related to post-deployment health (44%) were published in 2015. One large VA-funded RCT studied the impact of online expressive writing on readjustment difficulties among Veterans of Iraq and Afghanistan conflicts.^15^

#### Participants

Most studies had over 1000 participants (249/440, 57%). Of the 249 large studies, 71% utilized the VA electronic health record as a major data source. Thirteen studies enrolled clinicians or administrators as participants (e.g., a survey of VHA emergency department directors focused on capacity to meet the needs of female Veterans). Of the remaining 427 studies, 44% included only women, while 20% included less than 10% women.

#### Study Design

Most studies (398/440, 90%) utilized an observational research design such as a cohort, cross-sectional, or case–control design. Eight studies described the primary findings of RCTs, five of which were published since 2013. The two trials published in 2008^12^^,^ 14 were also identified in the previous review.^5^ Five percent (22/440) of articles were qualitative studies involving in-depth interviews or focus groups, nearly half of which were published in 2015. None of the included articles described significant patient or Veteran engagement in the study design or implementation.

#### Publication Year

**Fig. 3:**
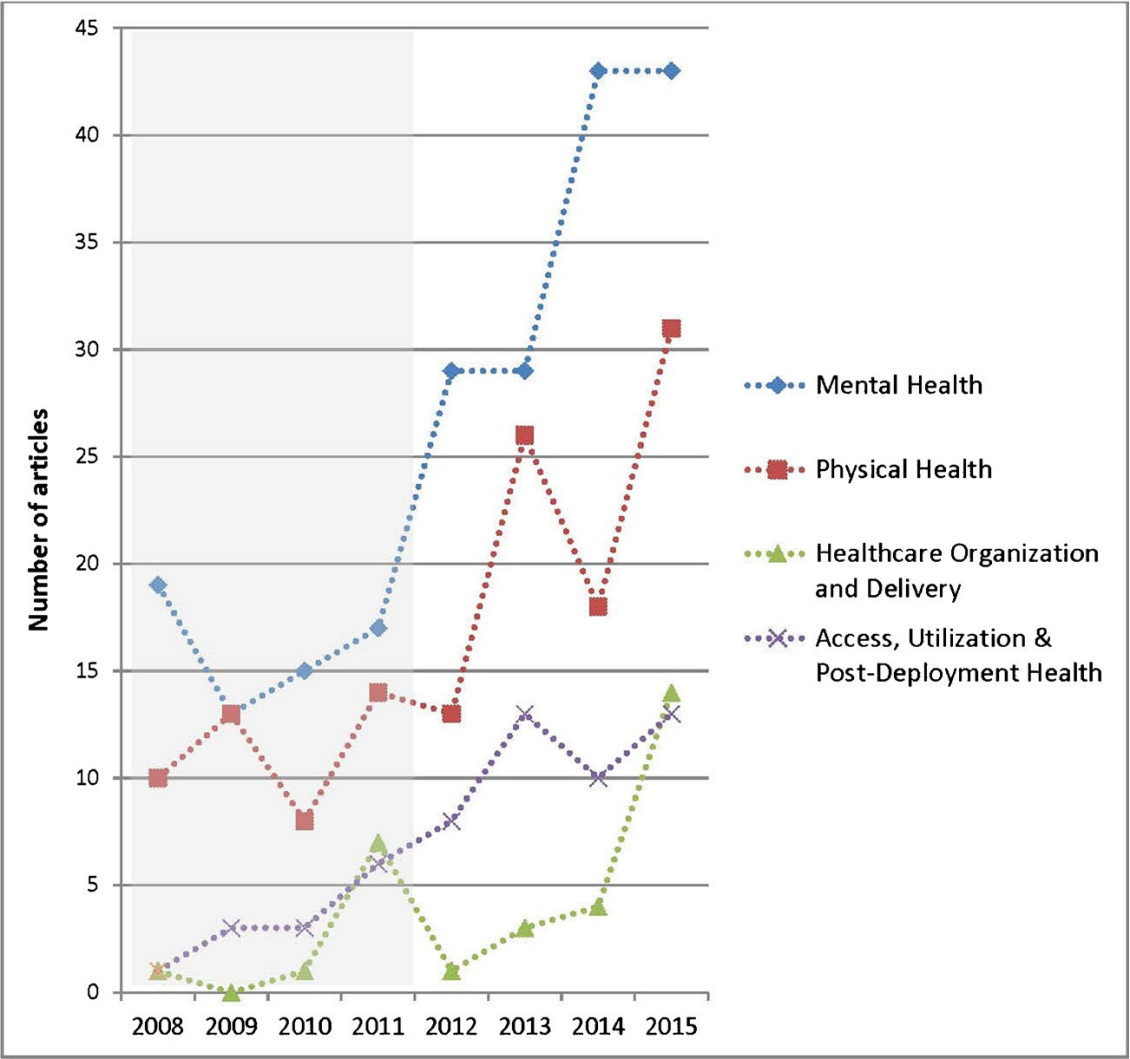
Number of Articles Published by Year and Healthcare Topic. * The VA Women’s Health Research Network was established in 2010 and published a priority research agenda in 2011.

The number of articles published per year grew over the 8-year review period (Fig. 3). From 2008 to 2011, 135 articles were published, whereas from 2012 to 2015 more than double that number (305 articles) were published. More articles were published in 2015 (101), than in 2008, 2009, and 2010 combined. Several infrequently studied healthcare topics prioritized by the WHRN in 2011^8^ grew rapidly thereafter, including reproductive health, healthcare organization and delivery, access and utilization, and post-deployment health. Two healthcare topics did not follow the pattern of increasing publications after prioritization by the WHRN: long-term care and aging, and prevention and screening. Long-term care and aging showed no change over time. Prevention and screening was the only topic with a drop in research over time, from 11 articles published during the first half of the review period to seven during the second half.

#### Funding Source

Overall, 69% of articles (302/440) reported VA funding. Less than 7% had Department of Defense (DOD) funding (29/440). Fifteen percent (65/440) reported funding from other governmental sources, such as the National Institutes of Health (NIH). A small number of studies reported foundation (24/440, 5%) or university (18/440, 4%) funding. Less than 2% (7/440) of studies explicitly stated that they were unfunded (all observational), and only four studies (4/440, <1%) reported industry (all pharmaceutical) funding. Some studies reported more than one funding source, but 20% did not specify any funding.

Funding sources varied somewhat by healthcare topic. Whereas around 80% of reproductive health (20/24), healthcare organization and delivery (25/31), and access and utilization (19/24) articles were VA-funded, only about half of prevention and screening (10/18), post-deployment health (9/18), and homelessness articles (6/12) were VA-funded. Two-thirds of articles (19/29, 66%) with DOD funding addressed mental health issues, while just under half of articles (29/64, 45%) with other governmental funding addressed physical health issues. Articles about post-deployment health (7/18, 39%) and homelessness (5/12, 42%) were most likely to not specify a funding source.

## DISCUSSION

Our evidence map describes the broad field of research related to female Veterans’ health and healthcare published between 2008 and 2015. The majority of identified studies were observational VA-funded studies, and nearly half were related to mental health conditions. We observed increased research in some priority topic areas, such as reproductive health, healthcare organization and delivery, access and utilization, and post-deployment health. However, we found few studies related to common chronic conditions seen in primary care and limited progress from observational to interventional research.

### Advances in Research Priorities

In 2011, the VA WHRN set forth an ambitious research agenda with six key topic areas: (1) mental health, (2) primary care and prevention (including primary care delivery), (3) reproductive health, (4) complex chronic conditions/aging and long-term care, (5) access to care and rural health, and (6) post-deployment health.^8^ We found evidence that four of these areas advanced considerably in subsequent years, as did the subsection of primary care related to healthcare delivery. Complex chronic conditions/aging and long-term care, and the remainder of primary care and prevention, did not show substantial growth and are addressed separately below.

Mental health articles continue to dominate the VA women’s health literature (47% of studies), consistent with the previous review (85/195, 44%).^5^ PTSD studies remain prominent but now represent only one-third of mental health research, compared with nearly half in the previous review.^5^ In contrast, sexual trauma and substance abuse have grown considerably as a proportion of mental health research.^5^ Research related to the delivery of comprehensive primary care for female Veterans shows evidence of coordinated growth, involving varied viewpoints (providers, Veterans, vulnerable subpopulations) and multiple methodologies (observational studies, qualitative studies, and an RCT). Several other topic areas (reproductive health, access to care, rural health, and post-deployment health) with little research at the outset of our study period have grown dramatically in number of publications since being named research priorities, with publication counts rising as much as seven-fold.

The VA WHRN has also emphasized research related to particular subpopulations of female Veterans. Returning Veterans of Iraq and Afghanistan conflicts make up one-third of living female US Veterans.^16^ Over one-fifth of included articles targeted Veterans of those conflicts, and three-quarters of those have been published since 2012. The majority of studies addressing LGBT Veterans, racial and ethnic minorities, and homeless Veterans have also been published since 2012.

The overall increase in publications in recent years can be at least partially attributed to VA-funded journal supplements in 2011,^7^ 2013,^17^ and 2015.^18^ The proportion of female Veterans’ health research that is VA-funded has also grown from 45% (studies from 1978 to 2004)^4^ to 60% (2004–2008)^5^ to 69% of studies in this review (2008–2015).

### Gaps in the Literature

We identified five primary gaps: research on common chronic disease topics, sex-specific results reporting, interventional study design, funding reporting, and Veteran engagement. First, several topics had surprisingly little research relative to their clinical prevalence—specifically, physical health topics in primary care and chronic disease, prevention and screening, and long-term care and aging. For example, we found no studies with a primary focus on hypertension though hypertension affects nearly 40% of middle-aged female Veterans in the VA and over 60% of those over 65.^3^ Controlling hypertension and other cardiovascular disease risk factors is critical for women, one in four of whom will die of heart disease.^19^ In addition, mental health topics most often encountered in primary care, including depression, anxiety, and postpartum depression, were largely absent from the literature. Depression is the most common mental health diagnosis among female Veterans at VA,^3^ including those returning from Iraq and Afghanistan.^20^ Evidence maps are primarily descriptive and our results do not directly address the causes or consequences of literature gaps. However, the stark disparity between the prevalence and significance of common chronic health conditions and the quantity of published research addressing those topics merits review.

We suggest that the apparent inattention to common chronic health conditions is primarily attributable to (1) the stage of existing evidence for most common conditions in primary care and (2) a lack of sex-specific results reporting for clinical research that includes female Veterans. For conditions such as hypertension and depression, decades of federally funded clinical research has defined best practices for healthcare. As a result, focusing ongoing research on health services delivery may be the most appropriate way to optimize the quality of care for female Veterans with these conditions. A 2008 VA Under Secretary for Health workgroup report on the provision of primary care to female Veterans highlighted the complexity of treating female Veterans with multiple comorbid chronic mental and physical health conditions, and identified fragmentation of care for general and gender-specific health concerns.^21^ Since 2008, most research related to common chronic conditions among female Veterans has addressed healthcare organization and delivery. For example, though very few articles in our sample primarily addressed depression, we found additional studies evaluating depression comorbid with physical health conditions and exploring integrated mental health and primary care delivery.

At least some research on common chronic conditions is being conducted with female Veterans, but the study results are not consistently reported by sex. We excluded over 350 articles that did not report sex-specific results. Though we did not extract study characteristics for excluded articles, a title search found 24 articles with the words “diabetes” or “depression” in the title (though only three had “hypertension” or “blood pressure,” and none had “anxiety”). The need for sex-specific reporting of scientific research results has been recognized by both the NIH^22^ and the Institute of Medicine,^23^^,^ 24 though multiple challenges related to study design, statistical analysis, and results reporting exist.^24^ VA has long required the inclusion of women in research,^8^ and encouraging sex-specific results reporting could expand the field of female Veterans health research and allow for future meta-analyses by sex.

Conducting interventional research among female Veterans has been challenging due to the small number of women at any one clinical site.^25^ We identified only eight published RCTs over the past 8 years. A simple search of excluded studies with the words “randomized trial” in the title revealed at least seven additional RCTs that included female Veterans but did not provide sex-specific results, and four more that included too few women to meet our criteria. Increasing the recruitment of women into existing VA trials and encouraging sex-specific results reporting could augment the field of experimental research related to female Veterans' health.

Reporting the source and role of funding is a quality standard for both experimental^26^ and observational^27^ research. Though 78% of studies identified at least one source of funding (a relatively high rate of funding compared to other medical fields),^28^^–^ 30 20% of included articles did not report a funding source, which likely represents unfunded research.^31^ Explicitly describing research as unfunded will help stakeholders allocate resources.

Finally, although several studies incorporated Veterans’ perspectives, they all adhered to a traditional model of women as study subjects rather than as research stakeholders or partners. Researchers are increasingly seeking to engage patients and community members in the development of study questions, selection of outcome measures, and interpretation of findings. Incorporating female Veterans’ voices in the production of future research will strengthen the relevance and credibility of that work.

## LIMITATIONS OF THIS EVIDENCE MAP

Due to resource limitations, we did not complete a dual review of all 2276 abstracts or 1184 full-text articles. As described in the methods section, we used a multifaceted approach to screen eligible articles and achieve consistent categorization.

An inherent methodological limitation of evidence maps is that they present a broad but relatively superficial description of the literature within a field. Beyond documenting the topics or methodologies we did not encounter, we cannot conclude which research questions have been sufficiently addressed versus which deserve additional attention. Advancing specific fields of research will require in-depth reviews of study quality and bias, as well as a synthesis of outcomes, all of which were outside the scope of this review.

## CONCLUSIONS

This large and varied body of research represents a growing evidence base that can be leveraged to improve the health of female Veterans. The VA is currently a leader in the field addressing military-related mental health conditions in women, such as PTSD. Recent research to improve the quality of primary care for female Veterans has been focused on the organization and delivery of care. As a nationally integrated healthcare system with a growing population of female Veterans and life span coverage, the VA is poised to be a leader in women’s health research. VA research and clinical stakeholders can use this evidence map to help direct the future of female Veterans’ health research.

## Appendix A

**Table 2:** Included References by Healthcare Topic

Healthcare Topics		Number of Studies	Reference
Mental Health *Total: 208 articles*	PTSD and trauma	71	32 ^–^ 102
	Military sexual trauma	37	103 ^–^ 139
	Mental health comorbid with non-mental health	23	140 ^–^ 162
	Substance abuse	20	163 ^–^ 182
	Multiple mental health diagnoses	16	20 ^,^ 183 ^–^ 197
	Suicide	13	198 ^–^ 210
	Intimate partner violence	9	211 ^–^ 219
	Disordered eating	5	220 ^–^ 224
	Depression and anxiety	4	225 ^–^ 228
	Reproductive mental health	4	229 ^–^ 232
	Serious mental illness	3	233 ^–^ 235
	Personality disorders	0	
	Other mental health topics	3	236 ^–^ 238
Physical Health *Total: 133 articles*	Reproductive health	24	239 ^–^ 262
	Prevention/Screening	18	12 ^,^ 263 ^–^ 279
	Long-term care/aging	13	13 ^,^ 280 ^–^ 291
	Cardiovascular disease	11	292 ^–^ 302
	Obesity	9	303 ^–^ 311
	Chronic pain	7	312 ^–^ 318
	Comorbid medical conditions	7	319 ^–^ 325
	Cancer	6	326 ^–^ 331
	Tobacco	6	332 ^–^ 336
	Traumatic brain injury	5	337 ^–^ 341
	HIV/AIDS	5	342 ^–^ 347
	Multiple sclerosis	4	348 ^–^ 351
	Diabetes	3	352 ^–^ 354
	Spinal cord injury	1	355
	Traumatic amputations	1	356
	Hypertension	0	
	Other medical conditions	13	357 ^–^ 369
Healthcare Organization and Delivery *Total: 31 articles*	Comprehensive and primary care delivery	16	14 ^,^ 370 ^–^ 384
	Mental healthcare delivery	9	385 ^–^ 393
	Emergency care delivery	3	394 ^–^ 396
	Virtual or telehealthcare delivery	3	397 ^–^ 399
Access, Utilization & Post-Deployment Health *Total: 57 articles*	Post-deployment health	18	400 ^–^ 417
	Barriers and facilitators of care	13	418 ^–^ 430
	Homelessness	12	431 ^–^ 442
	Healthcare utilization	11	443 ^–^ 453
	Rural healthcare	3	454 ^–^ 456
Other		11	457 ^–^ 467
TOTAL NUMBER OF INCLUDED STUDIES		440	
